# The push out bond strength of polydimethylsiloxane endodontic sealers to dentin

**DOI:** 10.1186/s12903-019-0867-5

**Published:** 2019-08-08

**Authors:** Kinley Dem, Yingfang Wu, Atipatsa Chiwanda Kaminga, Zhuo Dai, Xin Cao, Bingyu Zhu

**Affiliations:** 1 Centre of Stomatology, Department of Conservative Dentistry and Endodontics, Xiangya Hospital, Central South University, No.87 Xiangya Road, Changsha, 410008 Hunan China; 2 Xiangya School of Public Health, Department of Epidemiology and Health Statistics, Central South University, Changsha, Hunan China; 3 Department of Mathematics and Statistics, Mzuzu University, Private Bag 201, Mzuzu 2, Malawi

**Keywords:** Push out bond strength, AH plus, GuttaFlow 2, GuttaFlow bioseal

## Abstract

**Background:**

The purpose of this experiment was to assess the push out bond strength of Polydimethylsiloxane sealers (GuttaFlow 2 and GuttaFlow Bioseal by Colte’ne/Whaledent, Altstätten, Switzerland). AH Plus (Dentsply, DeTrey, Konstanz, Germany) was used as a reference material for comparison.

**Methods:**

Thirty root slices were prepared from the middle third of 10 mandibular premolars. Each slice was 1 ± 0.1 mm thick. Three holes, 0.8 mm wide each, were drilled on the axial side of each root slice. These holes were subjected to standardized irrigations and then dried using paper points. Finally, for each root slice, each hole was filled with exactly one of the following three root canal sealers: AH Plus, GuttaFlow 2 and GuttaFlow Bioseal. After all the holes were filled in that way, the root slices were stored on top of phosphate-buffered saline solution (pH 7.2) soaked gauze for 7 days at the temperature of 37 degrees Celsius. Then, for each root canal sealer on a root slice, the universal testing machine was used to measure the push out bond strength. The differences in push out bond strengths between the three sealer samples were assessed using the Friedman test, while the paired comparisons were assessed using Wilcoxon signed rank test with Bonferroni correction. All statistical tests were two-tailed and the significance level was set at the 5%.

**Results:**

According to the Friedman test the distributions of push out bond strengths of AH Plus, GuttaFlow 2 and GuttaFlow Bioseal were different (*P* < 0.05). Paired comparisons indicated that AH Plus had a significantly superior push out bond strength than GuttaFlow 2 and GuttaFlow Bioseal, while the push out bond strength of GuttaFlow Bioseal was significantly stronger than that of GuttaFlow 2 (*P* < 0.01).

**Conclusions:**

Based on these findings, AH Plus is a better root canal sealer than GuttaFlow 2 and GuttaFlow Bioseal.

## Background

A successful root canal treatment can be achieved when sealers are used to prevent the residual bacteria and endotoxins from crossing the root apex [1]. Nevertheless, after a chemical-mechanical preparation, some sealers may not be successful in preventing the foregoing infection [2, 3]. In this regard, it is necessary to identify an optimum sealing material that is capable of maintaining bond to the dentine wall, preventing the preceding infection, and resisting dislodgement of the filling.

Typically, push out bond strength determines the extent of resistance to the dislodgement of a filling material when applied to root canal dentine. In order to establish push out bond strength, a tensile load is positioned vertically to the long axis of the root till the filling is displaced [4]. Uregan et al. [5] indicated that push out bond strength showed better assessment of the bond strength than the conventional shear tests.

Although push out bond strength test might not be reliable in terms of representing clinical conditions of the sealers [6], currently this is the best adhesion test available [7]. Moreover, this test is easy to conduct, interpret and document unlike other many techniques, which may involve different core materials as well as various preparation procedures of root dentine [8–12]. While some push out bond strength methodologies used precise machine-made holes produced in the same dental slice [13–15], this study involved the latest push out bond strength methodology with standardized root canal structure artificially created [16].

Although there are a range of resins in endodontics and several kinds of sealers available, such as zinc oxide eugenol, glass ionomer cement, calcium hydroxide, and silicone based; there have been continuous developments in the quality of sealers. These developments are aimed at finding an ideal sealer that could make treatment more successful.

Meanwhile, silicone based group of sealers has shown excellent sealing ability because it is insoluble, expandable, and has excellent flow ability [7–9, 13, 17]. In the year 2012, GuttaFlow 2 (Coltene/WhaledentAG, Altstatten, Switzerland), a polydimethylsiloxane sealer, was introduced as an improved version of GuttaFlow. With reference to the manufacturer’s description, GuttaFlow 2 (GF 2) comprises gutta-percha powder of less than 30 μm in size, and micro-silver particles with a solubility of 0%. Studies conducted on GF 2 revealed good biocompatibility [9, 10] and sealing ability [11]. Later, in 2015, GuttaFlow Bioseal (Coltene/Whaledent AG, Altstatten, Switzerland) was introduced. It contains calcium silicate combined with gutta percha, unlike previous GuttaFlow sealers. According to the manufacturer’s description, the change in the composition of GuttaFlow Bioseal ensured that it should now have the ability to regenerate and heal tissues in the root canal. Although the biocompatibility and most of the physicochemical properties of GF2, GuttaFlow Bioseal (GFB) and AH Plus had been tested out and proven to be promising endodontic materials [12, 14], to the best of our knowledge no study had been conducted to test the push out bond strength of these root canal sealers and root dentine. Therefore, this investigation was conducted to find out which of these materials could be considered superior with regard to push out bond strength. Noteworthy, AH Plus (Dentsply DeTrey GmbH, Konstanz, Germany) is a widely used Epoxy resin-based root canal sealer with good physicochemical features and adaptability to the root canal walls [15], and a longstanding dimensional stability and lower polymerization stress [16]. Hence, it was frequently used for comparison with other sealers [18–20]. The null hypothesis to be tested is that the push out bond strengths of the three sealers is the same.

## Material and methods

### Sample selection and preparation

**Fig. 1:**
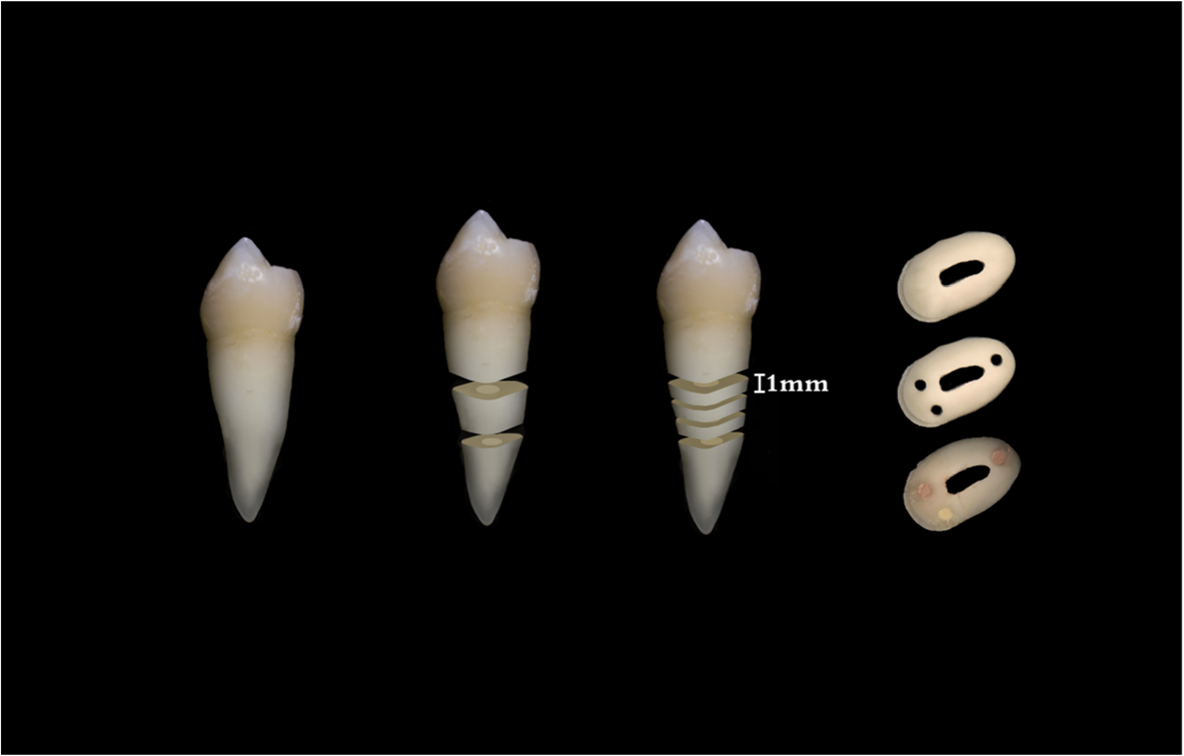
Photographic depiction of obtaining the root slice, then holes followed by filling with the endodontic test sealers

The local ethical committee of the Xiangya Hospital of Central South University approved this study (IRB [C] NO. 201706009). In addition, patients were requested if they could provide their teeth to be used as specimen. When they gave consent, they were asked to sign an agreement form to indicate that they were willing to provide their teeth for specimen. Following this, a total of ten lower first premolars that had been removed due to orthodontic treatment were collected. All collected teeth were verified using cone beam computed tomography (CBCT) before extraction to rule out calcified canals, caries, extra canals, open apices, cracks, and restorations. The teeth were immediately soaked in sodium hypochlorite 5.25% (Wei Zhen Yuan Co., Ltd., Fujian, CHINA) for 10 min. This was done to clean the soft and hard tissue debris. Later, the water-cooled diamond disc was used to cut off the coronal and apex sections of each tooth to obtain an intact middle third portion tooth. Then the three slices (1+/− 0.1 mm thickness, Fig. 1) were obtained using a low speed diamond saw (SYJ-160, MTI Corporation, Hefei, CHINA) with a diamond disc (125 mm × 0.35 mm × 12.7 mm, MTI Corporation, Hefei, CHINA) in constant water irrigation [21]. To confirm the final thickness of each slice, digital Vernier Caliper with accuracy of 0.001 mm (Avenger Products, North Plains, Oregon, USA) was used. A total of 30 dental slices were created following this procedure.

### Preparation of canal-like holes for push-out assay

Using a 0.8 mm cylindrical carbide bur, three canal-like holes, each to be filled with exactly one of the three materials, were drilled on each root slice to establish a fair comparison between the materials. A vertical drill stand (Dremel Workstation 220, Mount Prospect, Wisconsin, USA) was used to drill standardized holes parallel to the root canal amid continuous water irrigation. During this process, a minimum of 1 mm distance was maintained between the holes, external cementum, and the root canal wall [22].

After preparation, all the specimens were immersed in 2.5% sodium hypochlorite (NaOCl, Hubei Taichen Jianrui Pharmaceutical co., Ltd., Hubei, CHINA) solution for 15 min, and then rinsed in distilled water to counterbalance the NaOCl solution. The smear layer was removed by soaking the dental slices in freshly prepared 17% of Ethylenadiaminetetraacetic acid (EDTA, Haixi Hubei Co., Ltd., Hubei, China) for 3 min and subsequently in distilled water for a minute. Afterwards, dental slices were transferred to 2.5% NaOCl for 1 min. Finally, a wash with distilled water for 1 min was performed, and dried with paper points.

The three dental sealers: GF 2, GFB and AH Plus were randomly placed in the three holes of the root slice such that one sealer was exactly placed per hole. Using the manufacturer’s guidelines, the sealers were placed in the holes by vibrating gently so as to prevent bubble formation when placing the materials. Table 1 summarizes the composition of repair materials used. In the end, the samples were put on top of the moistened gauze soaked in phosphate buffered saline solution (PBS) (pH 7.2, Xiamen Science and Technology Co., Ltd., Xiamen, CHINA) at the temperature of 37 degrees Celsius (DNP-9160BS, Shanghai, CHINA) for 7 days to set the sealers [16].

**Table 1:** Compositions of the tested endodontic sealers

Sealer	Chemical composition
AH plus	Epoxy resin, calcium tungstate, zirconium oxide, aerosol, iron oxide, adamantine amine, bisphenol-A-diglycidyl ether, silicone oil
GuttFlow 2	Gutta-percha powder, polydimethylsiloxane, silicone oil, paraffin oil, platinum catalyst, zirconium dioxide, microsilver (Preservative), coloring
Guttaflow Bioseal	Gutta-percha powder particles, polydimethylsiloxane, platinum Catalyst, zirconium dioxide, calcium salicylate, Nano-silver particles, coloring, bioactive glass ceramic

### Push-out assessment

A plunger tip of 0.6 mm was positioned above a test sealer while avoiding the surrounding dental structures. Using a universal testing machine (318 10, MTS Systems Corporation, Eden Prairie, Minnesota, USA), pressure was applied in a coronal apical direction at the rate of 0.5 mm min^− 1^ till the sealer got dislodged. Real time software plotted the load × time curve while testing. The bond strength was measured and recorded in MPa^2^. At failure, the load (expressed in Newtons) was divided by the area of the bonded interface. Calculation of the adhesion area of the root canal sealer was done using the following formula: area = 2 *π* r × h, where *π* = 3.14, r = radius of the hole with the root canal sealer (0.4 mm), and h = material’s height (1.0 mm) ^3^ [22, 23].

### Data presentation and analysis

The normality of the push out bond strength data was verified by the Shapiro-Wilk test. When data are not normally distributed the *P* value of this test is less than 0.05, otherwise data are considered normally distributed. A comparison between the distributions of push out bond strength data for the three materials was conducted using one-way analysis of variance (ANOVA) with repeated measures, when data were normally distributed; else this was done using a nonparametric equivalent, Friedman test. In each case, if the distributions are proven to be different, pairwise comparisons are conducted. In this study, pairwise comparisons were conducted with Bonferroni correction. All statistical tests were two-sided and the significance level was set at the *α* =5%. Data were analyzed using the Statistical Package for the Social Sciences (SPSS) (SPSS 23; SPSS Inc., Chicago, IL).

## Results

**Fig. 2:**
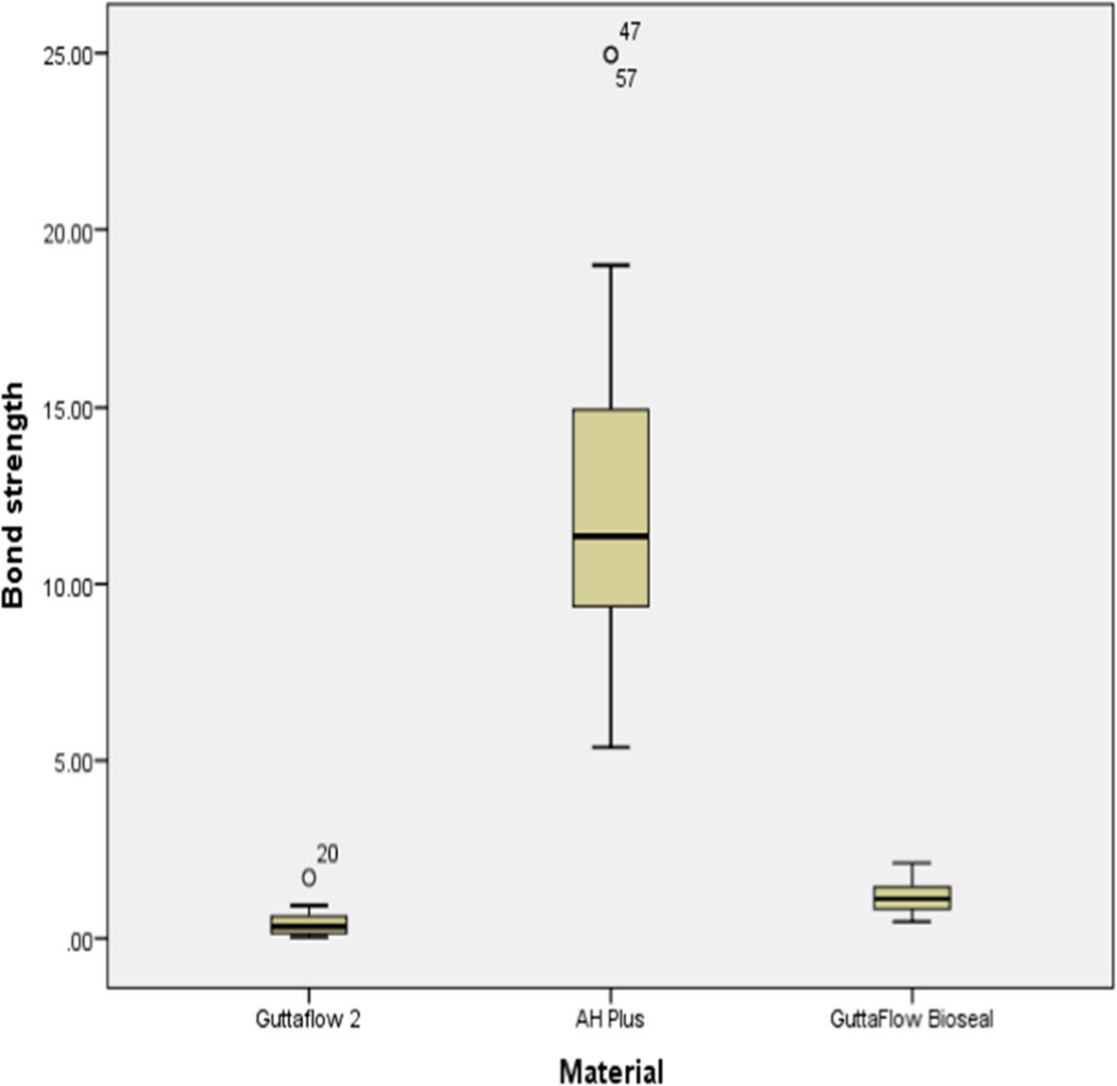
Box plots illustrating the push out values (minimum, median, maximum) of the tested sealers

Data were not normally distributed; hence Friedman test was used to compare the distributions of push out bond strength data for the three materials, and Wilcoxon signed rank test with Bonferroni correction was used to perform pairwise comparisons. A summary of the push out bond strengths of GF 2, GFB and AH Plus is given in Table 2. The results of the Friedman test (*P* < 0.05) indicated that the mean and median push out bond strength were highest for AH Plus, while GF 2 had the lowest. In particular, GFB had higher mean and median values than GF2 but lower than AH Plus. Besides, Table 3 presents the Wilcoxon signed rank test with Bonferroni correction, showing that GFB had significantly better push out bond strength value than GF 2 (*P* < 0.01). Overall, the push out bond strengths of the other two sealers were significantly lower compared to that of AH Plus. Figure 2 displays a graphical representation of the findings.

**Table 2:** Descriptive statistics for push out bond strength of the sealers

Sealers	No. Of samples	Mean	Standard Deviation	Median
GuttaFlow 2	30	0.43	0.37	0.33
GuttaFlow Bioseal	30	1.17	0.47	1.12
AH Plus	30	12.20	4.90	11.35

**Table 3:** Wilcoxon signed ranked test

Paired comparisons	Test statistics	*P* value
GuttaFlow2 vs AH Plus	− 1.933	<0.001
GuttaFlow Bioseal vs AH Plus	−1.067	<0.001
GuttaFlow2 vs GuttaFlow bioseal	−0.867	0.002

## Discussion

A root canal sealer must adapt well to the canal wall to create a strong sealer- dentine interface that can withstand mechanical stress [24]. The push-out bond strength test conducted in this study is relatively easy to perform, can replicate similar clinical conditions, has accurate specimen standardization, has minimal stress and has less technique sensitive [25–27]. It measures the material-dentine interfacial bond strength in all surfaces of the root canal [27, 28].

In contrast, other established methods for assessing push out bond strength involve the canal preparation, obturation and analysis of the different root sections of extracted teeth [20, 29]. These techniques have a disadvantage of causing inconsistent baseline measurements due to lack of proper standardization of the root canal anatomy. Moreover, according to the recent evolution in the methodological aspect recommended by Scelza et al. [22] and Silva et al. [30], this study used single dental slices each with three standardized holes to test the push out bond strength of three different sealers [22]. This was done to eliminate the complicating elements such as age of tooth, canal shape, scleroses, and micro-hardness in order to maintain a standardization of the comparisons. Additionally, a distance of 1 mm was maintained between any two holes, external cementum and the root canal surface to avoid fracture of the dental slice [22]. Standardized artificial holes of 0.8 mm in diameter were created to replicate the internal root canal anatomy [21]. With regard to the plunger tip size, Chen et al. [31] proposed that the size of the plunger tip should be 0.85 times smaller than the size of the filling material. Further, this researcher proposed that the position of the plunger tip must be closer to the diameter of the sealer. This allowed the plunger tip to concentrate the stress closer to the sealer dentine interface**.** Phosphate based solution (PBS) had been used as it was reported that calcium silicate enhances its push out properties in the presence of PBS moist environment [26]. Also, the cutting of a dental slice creates a coating of organic and nonorganic depositions known as smear layer, which may have bacteria and their by-products. Therefore, during instrumentations and obturation of the root filling materials, this smear layer could cause obstructions [32]. Thus, the use of EDTA and NaOCl was quite effective in eliminating the smear layer [16, 33].

Despite the preceding merits, the methodology of this study has several strengths and limitations. First, the creation of the standardized holes with burs may exclude the variables otherwise observed in clinical cases of root canal anatomy. Nevertheless, to control this type of failure, the artificial canals were only filled with root canal sealers, which may lead to stress concentrating on the sealers, but not on other materials, like gutta percha, as in other techniques. Second, although the use of a sealer with gutta percha would replicate more of a clinical situation, this procedure of using only a sealer would show the proper bond strength between root canal sealers and dentine [22, 30, 34].

In this study, the three sealers under discussion exhibited different bond strengths; therefore, the null hypothesis which stated that their bond strengths were the same is rejected. The ranking in ascending order of bond strength is given as follows: GF 2 < GFB < AH Plus. Accordingly, AH Plus was shown to have significantly the strongest push out bond strength, which is consistent with the results of some previously published studies [21, 24, 35, 36]. Therefore, this result could be owed to the development of a covalent bond. That is, a covalent bond forms epoxide rings when it is exposed to amino groups available in the collagen linkage, hence making the push out bond strength more resilient to pressure/stress [29]. In addition, AH Plus has been shown to have a long-standing dimensional stability and lower polymerization stress [19].

As regards the other two materials, they are similar in composition except that GF 2 contains micro silvers while GFB has nano silvers and calcium silicate. In comparison with AH Plus, GF 2 showed a significant lower level of push out bond strength. This was the case perhaps because AH Plus has better wettability than GuttaFlow [37]. This wettability of GuttaFlow against AH Plus was examined by checking the contact angle and surface area energy, whereby a short contact angle, and a larger surface free energy, presented higher wettability resulting in a better flow and interaction with the surface [37].

In addition, the lower push out bond strength of GF 2 might be due to the presence of silicone resin in its composition, which may cause an increase in the surface tension, hence making the flow of materials difficult, thereby causing it to have poor wetting effects [38]. These results suggest that chemical composition of a root canal sealer can have a considerable influence on the adhesion capability.

Furthermore, this study found that the latest root canal sealer, GuttaFlow Bioseal, has a significantly better push out bond strength than GF 2 but still less stronger than AH Plus. This observation may be explained by the fact that, when calcium silicate in GuttaFlow Bioseal comes in contact with the fluids, it forms a physical bond with the dentin surface by creating apatite interface deposits [12]. Thus, this may result in a higher push out bond strength [39, 40]. Also, an Environmental Scanning Electron Microscope (ESEM) analysis conducted by Gandolf et al. [12] presented that, with slight calcium release, low solubility and alkalizing activity of the calcium ions and phosphate ions stimulate the development of a superficial layer of calcium phosphate, which can fill out the voids and improve the sealing ability [40].

## Conclusions

The results of this study suggest that AH Plus exhibits higher push out bond strength when compared to GuttaFlow Bioseal and GuttaFlow 2. However, GuttaFlow Bioseal has slightly better push out bond strength than GuttaFlow 2.

## Data Availability

Data will be available on request from the corresponding author.

## Abbreviations

GF2: GuttaFlow 2
GFB: GuttaFlow Bioseal
NaOCl: sodium hypochlorite
PBS: Phosphate buffered saline solution
